# A Multi-Criteria Framework for Identification of Gully Developmental Stages Based on UAV Data—A Case Study in Yuanmou County, Yunnan Province, SW China

**DOI:** 10.3390/ijerph19138202

**Published:** 2022-07-05

**Authors:** Haimei Lin, Leichao Bai, Mingliang Luo, Zhicheng Wang, Dan Yang, Bin Zhang, Yebin Lin

**Affiliations:** 1 College of Life Science, China West Normal University, Nanchong 637009, China; linhm@stu.cwnu.edu.cn; 2 School of Geographical Sciences, China West Normal University, Nanchong 637009, China; luoml@cwnu.edu.cn (M.L.); wzc1990@cwnu.edu.cn (Z.W.); danyang_mh@cwnu.edu.cn (D.Y.); envgeo@163.com (B.Z.); forrest@cwnu.edu.cn (Y.L.); 3 Sichuan Provincial Engineering Laboratory of Monitoring and Control for Soil Erosion in Dry Valleys, China West Normal University, Nanchong 637009, China

**Keywords:** longitudinal profile, cross profile, gully erosion, gully developmental stage, multi-criteria decision analysis, dry-hot valley

## Abstract

Gully erosion is a common form of soil erosion in dry-hot valleys, and it often brings serious land degradation. A multi-criteria method integrating the characteristics of the longitudinal profile (LP), the cross profile (CP) and the knickpoints of gullies was applied to identify the development stage of gullies in Yuanmou County, Yunnan Province, in southwestern China. Firstly, based on the high-resolution data sources produced by an unmanned aerial vehicle (UAV), 50 gullies were selected as the typical ones in Tutujiliangzi and Shadi village. The LPs were extracted, and their morphological indices, information entropy and fitting functions were calculated. The morphological characteristics of the CPs and the presence or absence of knickpoints were recorded. The results show that the period of the gullies in Tutujiliangzi and Shadi is dominated by the deep incision period and the equilibrium adjustment period, which means that most gullies are in the period of the severe erosion stage. Among the gullies, 13 LPs’ morphological index is between 0.636 and 0.933, and the morphology of the LP presents an upward convex shape; the cross profiles are mainly V-shaped and U-shaped. Thirty-two LPs’ morphological index is between 1.005~2.384, which presents a slightly concave shape; the cross profiles are mainly repeated U-shapes. The remaining five LPs have a morphological index of 0.592, 0.462, 1.061, 1.344 and 0.888, respectively; the LPs of upstream and downstream are different. The LPs of the Tutujiliangzi gullies are nearly straight lines and slightly concave, while those of the Shadi village gullies are convex and nearly straight lines. The knickpoints and step-pools in Shadi village are more developed, while the gullies in Tutujiliangzi develop more rapidly. This study shows that in counties with similar conditions, these conditions such as temperature and precipitation, local topographic changes, soil properties and vegetation conditions have obvious effects on the development of gullies.

## 1. Introduction

It is a worldwide issue that soil erosion by water causes land degradation, particularly in arid and semi-arid regions [1,2]. Research shows that gully erosion is the main cause of soil degradation [3]. Gully erosion is an active modern geomorphological process and a component of the erosion system, it can predict runoff in rivers and ancient incised valley catchment areas [4]. Gully erosion is an important form of soil erosion, which has the characteristics of a large amount of erosion and a fast erosion rate in the rapid development stage, and is an important source of sediment in the watershed. During gully erosion, a large amount of sediment is carried away, resulting in serious soil and water loss. The sediment yield dominated by gullies accounts for 10~94% of the total sediment yield in the basin [5].

The morphological characteristics of gullies not only invert the law of erosion and sediment yield, but also record the law and process of gully development and evolution [6,7]. Research on gully morphology mainly includes the following aspects: (1) In terms of statistical parameters, scholars have explored the relationship between the morphological scale and erosion amount from the aspects of gully area, length, depth, density and volume [8,9,10,11,12], and analyzed the development direction of gullies and their influential factors. (2) In terms of morphological characteristics, the topographic and geomorphological characteristics of gully development were studied in-depth from the aspects of the gully head, gully node and shoulder line [13,14,15,16,17]. (3) In terms of profile characteristics, the research mainly focuses on characterizing gully profile morphology and predicting gully development trends through the topographic index [6,18,19]. Longitudinal profile analysis is more widely used in river geomorphology. For example, Jiang [20] analyzed the development mechanism and evolution law under typical tectonic conditions using Ivanov’s river longitudinal profile equation in the Jinsha River and other basins. Wu et al. [21] analyzed the valley longitudinal profile and calculated the information entropy of the Strahler curve. Zhao et al. [22] studied the development and evolution of the river longitudinal profile by fitting the shape of the longitudinal profile. Zhou et al. [19] studied the watershed in Japan’s hilly region, and the results showed that the longitudinal characteristics of the watershed played an important role in determining the topography of the entire watershed. Anderson et al. [23] determined the river geomorphic model based on the spacing of the channel units and the concavity of the water surface profile. In general, the representative studies on the evolution of valley topography include Davis’ “erosion cycle theory” [24], Horton’s law [25] and Strahler’s “hypsometric integral” [26], and so on. These research methods on geomorphologic evolution mainly describe the space–time evolution of large-scale erosion landforms, and whether they are suitable for the quantitative study of gully erosion in dry-hot valleys deserves further exploration. In summary, a large number of studies have involved the morphological characteristics of the longitudinal profile of the drainage basin; however, for the gully development area, how the morphological characteristics of the gully longitudinal profile reflect the evolution and development of the terrain, and whether there is a correspondence between the “erosion cycle theory” remain to be explored. Previous studies have divided the development process of erosion gullies into four stages, which are roughly the initial stage when the gully longitudinal profile is consistent with the original slope, the gully head incision stage, the trending equilibrium profile stage and the erosion stop stage [27]. Whether it is possible to study the development stages of gullies in a specific area, based on the above characteristics of the gully longitudinal profile and other characteristics, is worth further exploration.

Reviewing the existing research of gully erosion studies, the hot areas are concentrated in China, Japan, and the United States, and other places were also studied. Yuanmou is a typical dry-hot valley area in southwest China, where soil erosion is serious, and the gully system is well-developed and the shape is complex. The gully system has the characteristics of a large space scale, steep gully walls, active gully heads, which are usually convex and concave, and the headward erosion is intense [28]. The research on gullies is helpful to understand gully morphological characteristics and to provide the basis and reference for subsequent gully erosion and deposition for the Yuanmou dry-hot valley. In this article, the aims were to (1) extract the gullies and determine the gully developmental stage from three aspects: the longitudinal profile, the cross profile and the knickpoint, combined with the information entropy method and the mathematical function method, to construct a multi-criteria framework for the identification of gully development processes for use in the study area, and (2) compare and analyze the gully development characteristics in two typical areas of Shadi and Tutujiliangzi village, from the aspects of different soil properties and vegetation cover, in order to provide a scientific basis for the erosion development of gully landforms and the comprehensive management of soil and water conservation in this area.

## 2. Materials and Methods

### 2.1. Study Area

**Figure 1:**
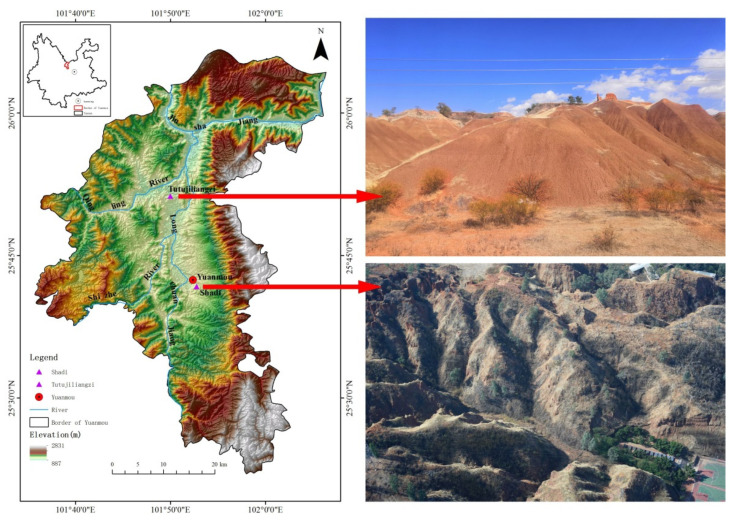
Location of the study area.

This article mainly takes Tutujiliangzi and Shadi village as the study area (Figure 1), which are located in the north and middle part of Yuanmou County, respectively. Yuanmou County is located in the lower reaches of the Longchuanjiang River, the first-order tributary of the Jinsha River. The parts below 1600 m in the basin are the typical dry-hot valley area, which is between 101°35′–102°06′ E and 25°23′–26°06′ N. The region belongs to the subtropical monsoon climate, with the characteristics of “hot and dry, concentrated precipitation, and distinct wet and dry seasons” [29]. The annual average temperature is 21.9 °C, the annual sunshine duration is long, with an annual average sunshine duration of 7.3 h/day, and the annual average precipitation is about 630 mm; precipitation in the rainy season accounts for about 90% of the whole year. In addition, the vegetation coverage rate in this area is low, causing serious soil erosion. In the geomorphic unit, they are, respectively, located in the Wumao Basin and the Yuanmou Basin within Yuanmou. The most exposed rock formations in this area are mainly metamorphic rocks, sandstones, mudstones and middle–late Pleistocene terrace sediments, and sedimentary rocks of fluviolacustrine can be seen everywhere [30]. The zonal soil in the dry-hot valley is mainly dry red soil, vertisol soil and red paleosol soil; sandy soil and clay are distributed in layers, and vegetation is rare. Below 1300 m above sea level, the basin is mainly barren hills and grassy slopes, mainly covered by sparsely growing shrubs and grasses. In addition to serious water and soil erosion in this area, the soil surface is eroded, which causes a decline in the soil fertility preservation ability and serious degradation [31].

### 2.2. Data and Research Methods

#### 2.2.1. Data Sources

**Figure 2:**
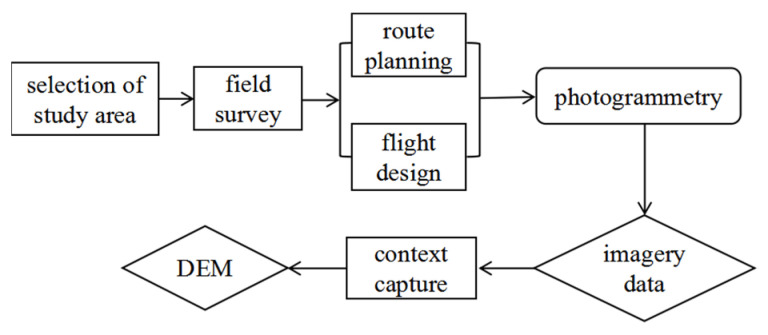
DEM data production process.

The data in this study were obtained by UAV photogrammetry. The measuring equipment was a flying horse D2000 multi-rotor UAV with a D-OP3000 tilt module of a SONY a6000 camera model. This type of UAV can realize high precision data measurement without using image control points, and its hovering accuracy is 1 cm + 1 ppm horizontally and 2 cm + 1 ppm vertically. To ensure that the shadows would not affect the image quality, we chose to fly from 10 am to 2 pm in cloudy weather, and the wind speed was either low or there was no wind [32,33]. Through field investigation, in order to avoid the influence of the terrain and to ensure the measurement accuracy, the flight heights of Tutujiliangzi and Shadi were set to 100 m and 110 m, respectively. During the acquisition process, the longitudinal overlap was 85% and the side overlap was 65%, which meets the standard of aerial photography in China. A total of 6095 aerial photographs were obtained. The aerial triangulation was calculated by Context Capture software. When there is no obvious cross or stratification in the inspection results, the three-dimensional point cloud with ultra-high density is generated by the operation. After removing the noise points, the DEM with a resolution of 0.1 m is established by creating an irregular triangle network. Figure 2 shows the production and processing stages of the DEM data in this experiment, including the field survey, route planning, flight design, aerial photography and the DEM data generated by Context Capture modeling.

#### 2.2.2. Information Entropy Analysis Method for Longitudinal Profile of Gully

The valley longitudinal profile has a specific shape at a specific moment and changes over time [20]. On the basis of the information entropy of the erosion basin, Ai [34] used the Ivanov curve to introduce the information entropy from the erosion basin into the general watershed:

(1) $$h=H{\left(\frac{l}{L}\right)}^{N}$$

where h and l are, respectively, the height and the horizontal distance from a point on the gully profile to the gully outlet; H and L are, respectively, the height and the horizontal distance between the gully head and the gully outlet, and N is the morphology index of the gully longitudinal profile. When N < 1, the shape of the valley longitudinal profile is an upwardly convex curve; when N = 1, it is a straight line; and when N > 1, it is a downwardly concave curve. 

According to the above equation, the information entropy of the longitudinal profile of the valley is established as [34,35]:

(2) H(N)=ln(1+N)−N(1+N)

The relationship between H(N) and the evolution of the gully longitudinal profile is: H(N) < 0.193, the gully is in the deep erosion period; H(N) = 0.193, it is in the transition period; and H(N) > 0.193, it is in the isostatic adjustment period; as the H(N) value increases, it enters the equilibrium profile stage.

The information entropy H(N) of the gully longitudinal profile describes the development and evolution of the gully in the basin after crustal uplift, which represent the time-process characteristics of the gully branch system in the basin system [21]. Combined with the development status of the erosion gully, when N < 1, the erosion gully roughly corresponds to the cutting stage of the gully head; when N > 1, the erosion gully corresponds to the equilibrium profile stage; and as the value of N increases, the erosion gully corresponds to the stop development stage. Therefore, based on the previous research, this article integrates the gully longitudinal profile morphology index and the gully longitudinal profile information entropy method, and applies the method of characterizing the development of the valley to the evolution and development of the gullies; Table 1 characterizes the development stages of the gullies. Due to the initial stage of gully development, the longitudinal profile is basically consistent with the original slope shape, and its shape cannot be well judged through longitudinal profile experiments; an appropriate division method has not yet been found to explain it temporarily.

#### 2.2.3. Longitudinal Profile Fitting Function

The river longitudinal profile can directly reflect the characteristics of the longitudinal profile. Four regression equations—linear, exponential, logarithmic and power functions—are generally used to quantitatively describe the relationship between some internal factors (such as flow, channel particle size, sediment transportation, etc.) and the river longitudinal profile [36,37,38]. The results show that under the relatively stable conditions of tectonic activity and moderate climate change, the concave degree and morphological changes of the river longitudinal profile reflect the evolution process of river; the corresponding evolution sequence is linear function → exponential function → logarithm function → power function [22,39]. The specific evolution process is as follows: after the terrain surface is uplifted by the tectonic movement, the initial concave degree of the river longitudinal profile is small and close to a straight line, which can be fitted by a linear function; with the strengthening of headward erosion, materials in the middle and upper reaches are transported downstream and accumulated, the concave degree of the river longitudinal profile becomes larger, which can be fitted by an exponential function; then, due to erosion gradually tending toward equilibrium, the river longitudinal profile can be fitted by a logarithmic function; and if the river flow or sediment transportation increases, the concave degree of the river longitudinal profile increases rapidly, which can be fitted by a power function.

Considering that during the development of erosion gullies, the longitudinal profile morphology has periods of convex upward curves, gully head undercutting or knickpoint occurrence, equilibrium adjustment and equilibrium profile, this study used the fitting function analysis method of the river longitudinal profile for reference.

#### 2.2.4. Multi-Criteria Decision Analysis Technique

**Figure 3:**
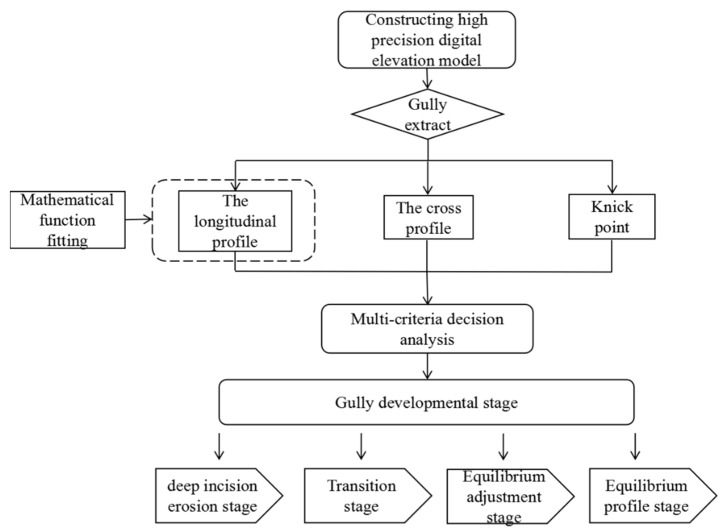
Methodological framework.

Multi-criteria decision making is a systematic method that takes the relevant influencing factors of decision-making objectives as the criteria for comprehensive analysis and finally obtains the decision-making scheme [40,41,42]. In this study, the longitudinal profile, the cross profile and the knickpoint that can well describe the gully developmental state in the gully development process were selected. According to the field observation and the actual situation, the position of the gully head was determined first, so as to determine the position of the gully. From the gully head to the gully outlet, the profile along the line in the gully bottom is the longitudinal profile, which can reflect the elevation change of each point along the path from the gully head. The gully longitudinal profile shape can reflect the evolution and development of the terrain and describe the change of the gully landform. The gully cross profile is a representation profile selected at a suitable position according to changes in the topography and channel width. The gully cross profile shape is important to understanding the relationship of the gullying process, the landforms, land use and erosional features [43]. According to the change of the longitudinal profile slope, the gully knickpoint is the break point of the slope change position; the knickpoint can reflect the gully retrogressive erosion degree and gully morphological characteristics [44]. The development stage of the gully was analyzed by using the multi-criteria decision analysis method in this article (see Figure 3).

## 3. Results

### 3.1. Morphological Characteristics of Longitudinal Profile

**Figure 4:**
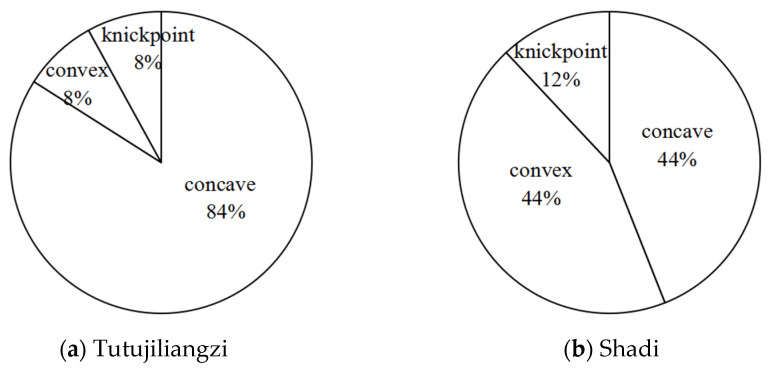
Percentage of longitudinal profile morphology.

This article extracted 25 gullies in Tutujiliangzi and 25 gullies in Shadi. The gully numbers of Tutujiliangzi are T1, T2, …; the gully numbers of Shadi are S1, S2, …. The area (A), length (L), morphology index of the longitudinal profile (N), information entropy of the gully longitudinal profile (H(N)), shape of the gully longitudinal profile and optimum-fit function are listed in Table 2 and Table 3; the best fitting functions are marked with an asterisk. The proportion of longitudinal profile morphological numbers of Tutujiliangzi and Shadi are shown in Figure 4a,b, respectively.

The morphological index and information entropy of the gully profile can reflect the evolution trend of the eroded landform. As shown in Table 2: (1) The maximum N value of Tutujiliangzi is 2.384, the minimum is 0.462 and the average is 1.337; the maximum information entropy H(N) is 0.515, the minimum is 0.064 and the average is 0.275. (2) The N values of four gullies (T4, T5, T13 and T17) are less than 1 and the information entropy is less than 0.193; it can be judged from these numerical values that these four gullies are in the deep incision erosion period. The longitudinal profiles of T13 and T17 are convex; the morphology of the longitudinal profiles of T4 and T5 is convex in the downstream direction and concave in the upstream direction. The N value of 21 gullies is greater than 1, and the information entropy is greater than 0.193, which shows that the 21 gullies are in the equilibrium adjustment period, indicating that the gully undercutting erosion is weak, and the lateral erosion is strengthened. The larger the N value is, the greater the degree of concavity, and the gullies gradually develop to the stable equilibrium profile. (3) As can be seen from Figure 3, in Tutujiliangzi, the concave gullies account for 84% of the total, and the convex gullies and the gullies with knickpoints account for 8% of the total, respectively, indicating that overall, the gullies in this area have developed from a sharp downward stage to an equilibrium profile stage.

It can be seen from Table 3 that: (1) The maximum N value of Shadi is 2.210, the minimum is 0.636 and the average is 1.222; the maximum information entropy H(N) is 0.458, the minimum is 0.104 and the average is 0.223. (2) The N values of 12 gullies are less than 1, most of them are close to 1, the information entropy is less than 0.193, and the longitudinal profiles are convex and nearly linear; among them, the shape of the longitudinal profile of the S12 gully is straight in the upstream direction and convex in the downstream direction. The N values of 13 gullies are greater than 1, the information entropy is greater than 0.193, and the longitudinal profiles of these 13 gullies are concave; among them, the morphology of the longitudinal profiles of S6 and S11 are as follows: S6 is straight in the upstream direction and convex in the downstream direction, and S11 is straight in the upstream direction and concave in the downstream direction; lateral erosion prevailed at this time, and then gradually developed into an equilibrium profile. (3) As shown in Figure 3, Shadi is dominated by convex and concave gullies, accounting for 44% of the total, respectively, and the gullies with knickpoints accounting for 12%. The results show that the gullies in the downward stage and equilibrium profile stage are mainly in Shadi; the development rate is slower than that of Tutujiliangzi.

### 3.2. Function Fitting of Gully Longitudinal Profile

Four mathematical functions—linear function, exponential function, logarithmic function and power function—were used to fit 50 longitudinal profiles of gullies in the two study areas, respectively. The main basis for judging the best mathematical fitting function is as follows: firstly, referring to the discriminant coefficient (R^2^) of the statistical regression between the mathematical function and the actual gully longitudinal profile; and secondly, the visual judgment of the coincidence degree between the actual gully longitudinal profile and the concave curve of the mathematical function. As can be seen from Table 2 and Table 3, there are 25 gullies in Tutujiliangzi, of which 12 gullies can be fitted by the exponential function, 8 gullies can be fitted by the power function, 3 gullies can be fitted by the linear function and 2 gullies can be fitted by the logarithmic function. In addition to the logarithmic function of T3, the linear function of T4 and T13, and the exponential function of T17, the best function fitting degrees of the rest of the gullies are all above 0.9. Among the 25 gullies in Shadi, 14 gullies can be fitted by the linear function, 3 gullies by the exponential function, 4 gullies by the logarithmic function and 4 gullies by the power function; except for the linear function fitting of S10, the best function fitting degrees of the rest of the gullies are above 0.9.

**Figure 5:**
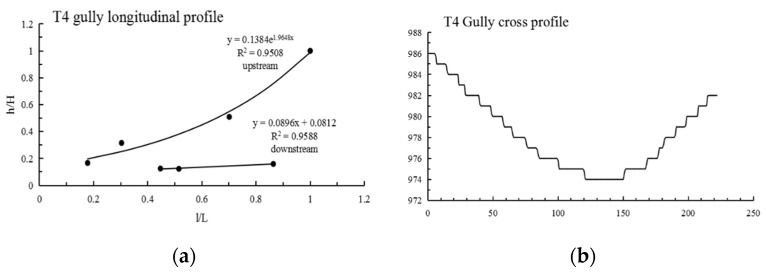
Longitudinal and cross profile fitting diagram of T4.

**Figure 6:**
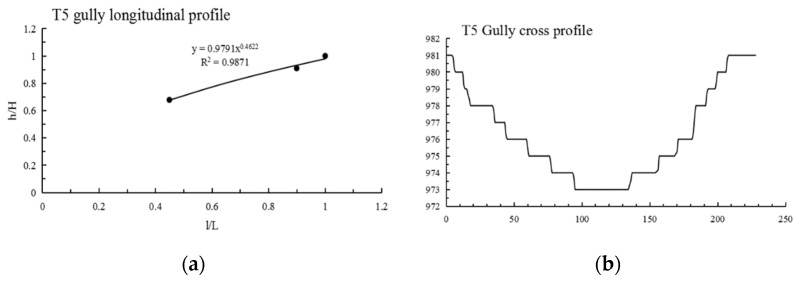
Longitudinal and cross profile fitting diagram of T5.

**Figure 7:**
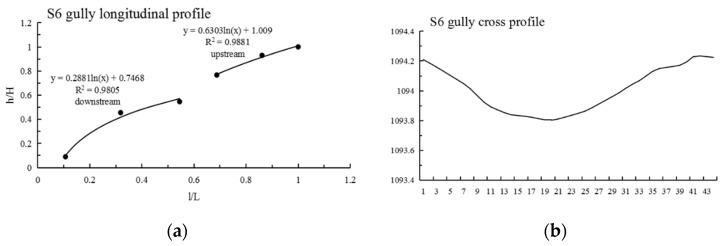
Longitudinal and cross profile fitting diagram of S6.

**Figure 8:**
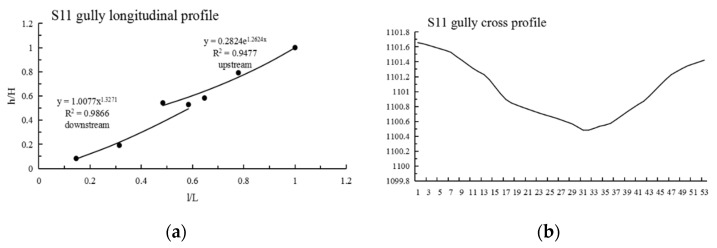
Longitudinal and cross profile fitting diagram of S11.

**Figure 9:**
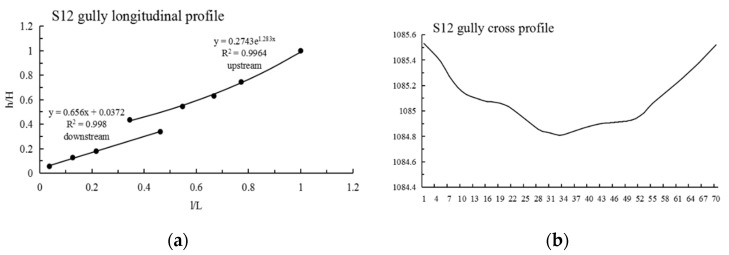
Longitudinal and cross profile fitting diagram of S12.

The mathematical function fitting of some gullies is very close to the actual gully longitudinal profile curve, and the gap between the values is very small; that is, the determination coefficient (R^2^) of some gully fitting functions is very high, but the mathematical function with a high R^2^ value is not necessarily consistent with the actual gully profile morphology, such as in T8, T10, S7, S8 and S9. Therefore, it is necessary to refer to the actual gully longitudinal profile shape for judgment, and at the same time, select the function with the highest degree of fitting with the actual gully profile shape as the best fitting function. After the above identification, the longitudinal profiles of the 25 gullies in Tutujiliangzi were found to be nearly straight and slightly concave as a whole, while the longitudinal profiles of the 25 gullies in Shadi were found to be convex and nearly straight as a whole. The function model shows that the gullies in the study area are in a stage of intense erosion. The fitting curve of some gullies comparing the elevation and the slope of the gully, combined with the gully parameters and the characteristics of the longitudinal and cross profile curves, revealed that this is due to the change of local topography on the actual gully longitudinal profile, and its slope value suddenly increases or decreases, resulting in the occurrence of knickpoints or step-pools, so that the entire gully longitudinal profile presents a compound profile, and its upstream and downstream profiles need to be fitted with two different functions, such as in T4 (Figure 5), T5 (Figure 6), S6 (Figure 7), S11 (Figure 8) and S12 (Figure 9). The upstream and downstream longitudinal profiles of T4, T5, S6, S11 and S12 are fitted with different functions. Due to the short length of T5, the piecewise fitting effect is not obvious.

### 3.3. Knickpoint

**Figure 10:**
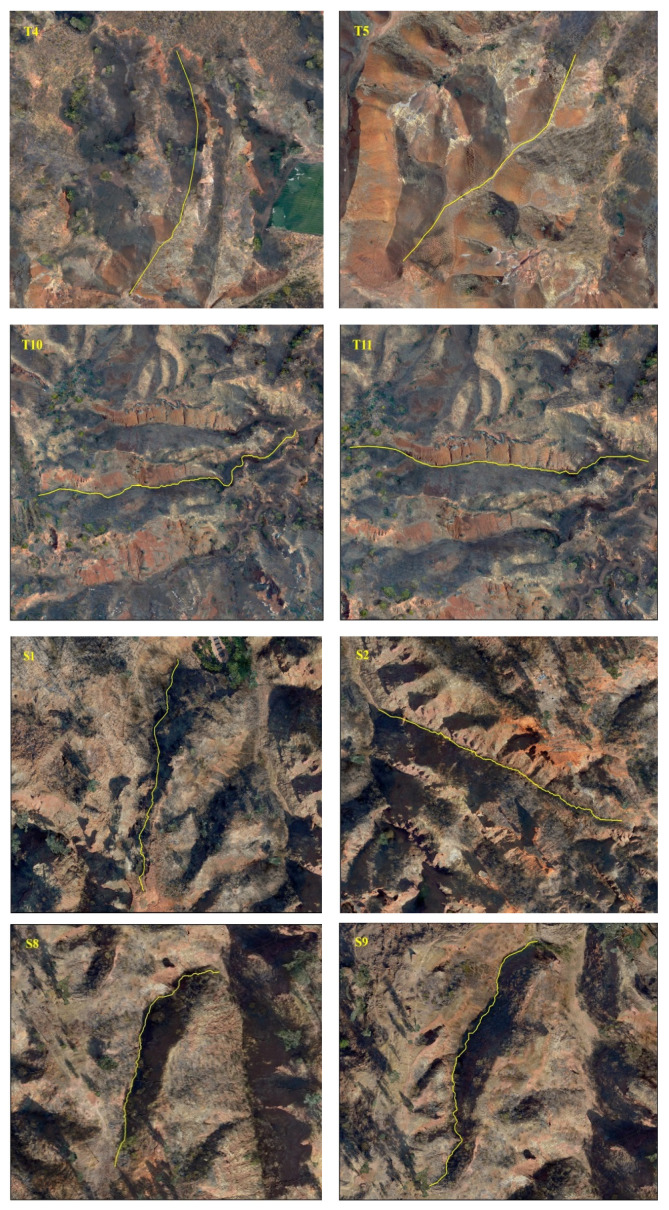
The gullies’ locations.

In the development process of a gully, when the erosion base level drops, a gully will cut down from the source near the erosion base level, and at the place where the erosion reaches, a slope turning point appears on the longitudinal profile of the gully bed, which is called a knickpoint [45,46]. The height of the gully bed on both sides of a knickpoint is obviously different from that on the ground; it is a kind of tectonic microgeomorphology phenomenon with its own specific formation and evolution law [47]. The development of the deep incision stage of an erosion gully develops rapidly on homogeneous and loose soil, such as loess, but slowly in the presence of hard rocks, because it is easy for hard rock to cause knickpoints in the gully bed or in the gully outlet. In this case, the gully is prone to produce multistage characteristics, that is, the upstream and downstream directions of the gully have different developmental stages. The partial gully location map can clearly show the location of the knickpoints (Figure 10). Knickpoints exist in the T4 and T5 gullies, and the S6, S11 and S12 gullies.

The above analysis results show that the 50 gullies in the Tutujiliangzi and Shadi area are mainly in the deep incision period and the equilibrium adjustment period; that is, they are in the stage of gully head cutting with strong erosion and in the equilibrium profile stage, and as a whole, they are in the active stage of development. According to the research, the average annual headward erosion rate of a gully in Yuanmou County is about 50 cm/a, the maximum is 200 cm/a, the gully density is 3.0–5.0 km/km^2^, the maximum is 7.4 km/km^2^, and the soil erosion modulus is up to 1.64 × 104 t/(km^2^·a) [5]. This is consistent with the conclusion that gully development is in an active stage. 

## 4. Discussion

### 4.1. The Indicating Effects of Longitudinal Profile Morphological Index and Longitudinal Profile Information Entropy

**Figure 11:**
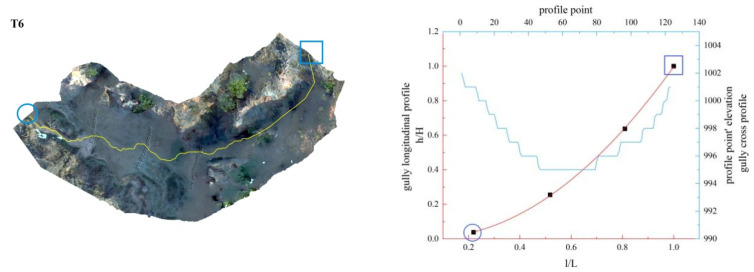
Gully shape and profile fitting diagram of T6.

**Figure 12:**
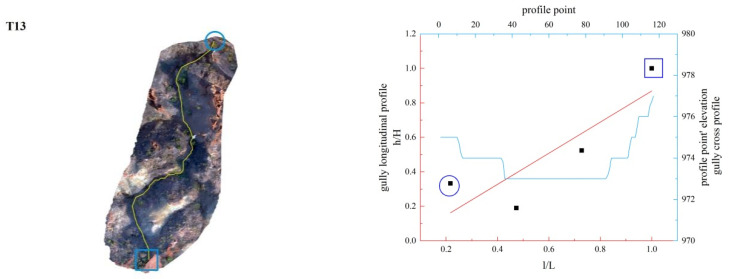
Gully shape and profile fitting diagram of T13.

**Figure 13:**
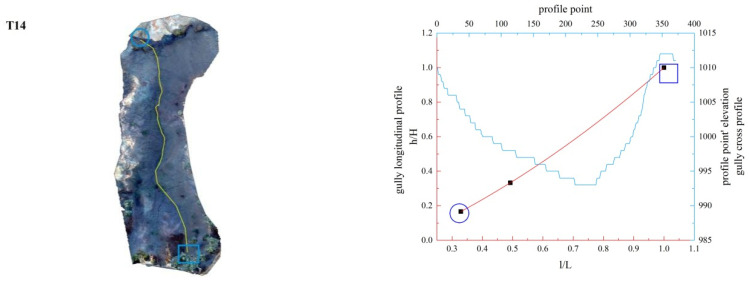
Gully shape and profile fitting diagram of T14.

**Figure 14:**
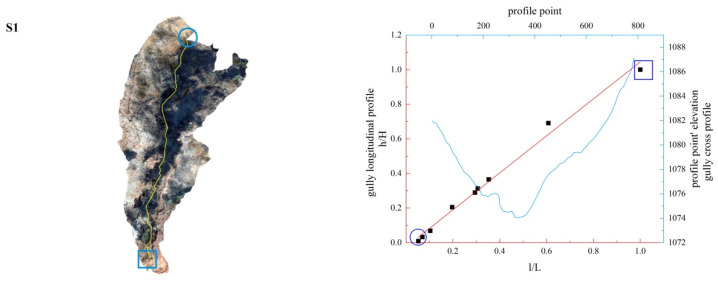
Gully shape and profile fitting diagram of S1.

**Figure 15:**
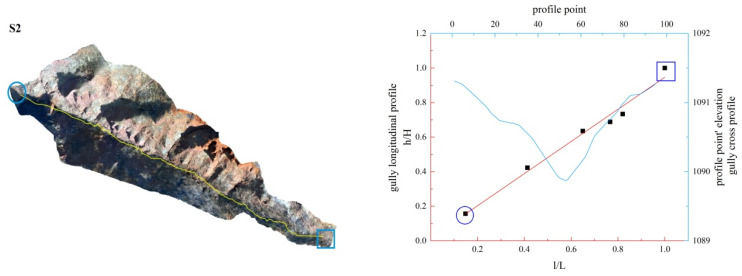
Gully shape and profile fitting diagram of S6.

**Figure 16:**
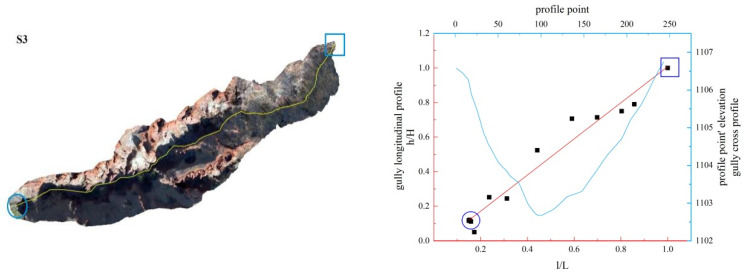
Gully shape and profile fitting diagram of S3.

In this study, the longitudinal and cross profiles of some typical gullies as well as the positions of the gully head and the gully outlet are listed (Figure 11, Figure 12, Figure 13, Figure 14, Figure 15 and Figure 16). Among them, the rectangular represents the gully head, and the circle represents the gully outlet. Comparing the fitted gully profile with the actual profile can more intuitively reflect the gully morphological characteristics. As can be seen from the figures, except for T13, the fitting profile of several gullies is basically consistent with the actual profile. The profile point fitted by T13 conforms to the actual profile distribution. The straight profile shows U-shaped and V-shaped cross profiles. Through the calculation of the slope of the gully, it was found that the slope at the gully head becomes steeper locally and the downcutting is obvious, which makes it easy for the gully head to form a water drop, and with strong erosion, sediment began to accumulate at the gully outlet. The concave profile shows a complex U-shaped cross profile; the cutting at the gully head stops and a large amount of sediment is generated at the gully outlet. These characteristics can be interpreted as follows: In the process of gully development, from the beginning to the end of the cycle, the gully mouth segment first began to cut down, forming a deep V-shaped valley. At the early stage of development, the headward erosion was very strong and the gully formed a convex profile [48]; the gully cross profile easily formed a V-shaped or U-shaped profile under intense erosion. With the weakening of retrogression erosion, gully development was dominated by deep and wide development; that is, the longitudinal and transverse erosion was intensified. At this time, the gully developed towards uniform linear and concave profiles [48], and the material accumulated at the bottom of the gully was washed away, forming a U-shaped cross profile [43]. When gully erosion gradually slowed down, the gully longitudinal profile eventually developed into an equilibrium profile, in which erosion and accumulation were balanced; that is, at the beginning, the convex profile was the most convex, then tends to become a straight line, and then became concave and developed to the equilibrium profile, with a gradually increasing concave degree [20]. Combining the image map and the morphology of the gully profile, it can be concluded that gullies in different development stages have different characteristics. The conclusions listed in Table 4 were reached by analyzing the characteristics of the longitudinal and cross profile, the shape of gully head and the location of the knickpoints.

Among the 50 gullies in the study area, the longitudinal profile morphological indices of 13 gullies are between 0.636 and 0.933, which are in the stage of deep incision erosion. At this stage, the downward erosion of the gully head intensifies and longitudinal erosion predominates. At this time, the longitudinal profile of the gully is inconsistent with the original slope shape; there are multisegment characteristics, which easily produce step-pools and knickpoints, the cross profile is V-shaped or U-shaped and the longitudinal profile is convex. Through the judgment of the fitting function, it was found that the characteristics of the fitting function conform to the actual situation. The longitudinal profile morphological indices of 32 gullies are between 1.005 and 2.384, which are in the stage of equilibrium profile; the gully longitudinal profile is close to the equilibrium profile, showing a slightly concave shape, affected by the eroded basement, downward erosion is no longer intense, and the lateral erosion is dominant, and the knickpoint is gradually approaching the gully head. The longitudinal profile morphological indices of five gullies are 0.592, 0.462, 1.061, 1.344 and 0.888, respectively. The upstream of T4 is in the equilibrium adjustment period and the downstream is in the deep incision erosion period; T5 is in the deep incision erosion period; the upstream of S6 tends toward the equilibrium adjustment period and the downstream is in the deep incision erosion period; the upstream of S11 tends toward the equilibrium adjustment period and the downstream is in the equilibrium adjustment period; and the upstream of S12 tends toward the equilibrium adjustment period, while the downstream is in the deep incision erosion period.

In the evolution of eroded watershed topography, Strahler used the hypsometric integral (HI) method to quantitatively study the geomorphic development stage; that is, in the early stage of watershed development, the erosion rate was fast and the HI value was large, but with the development of basin geomorphology, the erosion rate slowed down and the HI value gradually decreased [26]. The area-elevation integral values of 50 gullies were calculated and the results showed that the gullies’ area-elevation integral values in the deep incision erosion period were between 0.46 and 0.72, which were relatively large. The gully area-elevation integral values of 32 gullies in the equilibrium adjustment period were between 0.36 and 0.82; the value fluctuated greatly but was small on the whole. From the analysis results, it can be seen that there is a certain correlation between the longitudinal profile morphology index, the longitudinal profile information entropy and the hypsometric integral value. The gullies in the equilibrium adjustment period and the equilibrium profile period need to be further analyzed according to the actual situation.

In order to improve the accuracy of the experimental results, this study conducted a comparative analysis with DEM data with a resolution of 12.5 m. The results showed that the higher resolution data had a significant effect on improving gully morphological expression and the clarity of the gully development area. The high-resolution data are more effective for removing shadows and vegetation points in the gully path, and the fitting results are more in line with the actual situation. Erosion and runoff formation are highly scale-dependent processes and different scales of gully have different erosion effects. The main research object of this experiment was the gully, the effect of the rill and ephemeral gully extraction needs to be improved, and the comparative analysis under different spatial scale conditions needs more research. For extracting gullies with shorter lengths or smaller areas, high-resolution data are needed, and in the future, higher precision data will be obtained according to the actual situation of the study area, and this experiment will be improved. 

In this study, the gully development stages were analyzed by describing the gully longitudinal profile, the cross profile and the knickpoint positions, by quantitative and qualitative methods. Based on 50 gullies in the dry-hot valley of Yuanmou, a multi-criteria framework was established to classify the gully development stages. The information entropy analysis method for the longitudinal profile of the gully was used as the criterion for dividing the gully development stages, which was very effective in distinguishing deep and balanced gullies. In previous studies on gully evolution, Wu quantitatively divided the gully evolution state of a small watershed, according to the landform information entropy of Ai [21]. Yan used the hypsometric integral method to divide the erosion stages of gullies [49]. Different standards are adopted for different studies because geomorphology is a very complex process, which is affected by the natural environment and human activities. Therefore, the standards for dividing geomorphological development stages should be adjusted according to the specific conditions of the study area. The division results obtained in this study may have certain limitations in the research of other regions. How to establish the division standard for gully development stages suitable for most regions is still our ongoing work.

### 4.2. The Influence of Environmental Conditions on Gully Development

Previous studies have shown that the morphological development of gullies depends on human activities [50,51,52], such as farming, vegetation restoration and other natural factors such as soil properties, land use, tectonic activity, vegetation cover, climate and rainfall, etc. [53,54,55]. In this article, soil properties and vegetation coverage were compared and analyzed according to the local topographic differences in the two study areas.

(1) Soil properties

Soil properties can change the erosion rate and material transport by affecting slope runoff and infiltration, thus affecting gully longitudinal profile morphology [56]. Quaternary loose sediments have accumulated in the Yuanmou basin. The strata in Tutujiliangzi and Shadi belong to the Quaternary Pleistocene. The rocks are mainly gravel, clay and sandy clay [57], and coal or lignite and volcanic rocks are intercalated in the local rock layers. Such lithology has a great influence on the mechanical composition of the soil, resulting in loose soil lithology, a loose texture and easy erosion [43,58]. The study area is mainly composed of dry red soil, vertisol and red paleosol. The soil properties of Tutujiliangzi are relatively uniform and mainly composed of vertisol. The mechanical compositions of this kind of soil are mainly sand and clay, and such particle size components make the soil hard during drying, but it collapses easily after being washed by rain, and has strong collapsibility [59,60]. This feature can cause soil disintegration and loss in a short time in the rainy season, with the bottom of gullies constantly eroding downward, and at this time, deep incision erosion is intense downstream of the gully. The soil properties of Shadi are complex, and mainly composed of dry red soil and red paleosol. These two types of soil, which are widely distributed in Shadi, have a reddish soil color, a deep degree of weathering, and are extremely hard when dry. However, with the erosion from running water, the vertical joints of the soil develop and expand to form large cracks, which easily cause collapse and form sink holes, falling caves and washing holes, etc. [61] Due to the inhomogeneity of dry red soil, obvious stratification appears in the strata. The topsoil has strong corrosion resistance, there are hard rock strata in the middle which hinder the development of gully erosion, and obvious bulges are prone to occur in the gully during development; the lower soil lithology is loose and easily eroded. In addition, in Jinlei village of Yuanmou County, which is also dominated by vertisol, most gullies are in the active stage of rapid development, which is different from the developmental stage of Tutujiliangzi. In Maoyi village, which is dominated by dry red soil, gully development speed is relatively slow, which is similar to the situation in Shadi.

(2) Vegetation

Short-time heavy rainfall is the main form of rainfall increase in the Yuanmou dry-hot valley area [62], and sediments are transported and accumulated by rainwater in a short time [63,64]. Vegetation can effectively trap rainfall, slow down surface runoff [65,66] and increase soil erosion resistance, thereby inhibiting gully development and improving gully stability. The influence of human activities on vegetation change in the Yuanmou dry-hot valley has been remarkable since the ecological restoration work was developed. Gully activity is related to the coverage of shrubs and herbs, and there are usually artificial shrubs and other plants at the gully head [30]. Herbaceous plants often grow at the bottom of the gully, where they accumulate after decaying and form a humus layer [60]. The vegetation keeps the soil in the gully covered by the canopy and humus, which significantly moderates the intensity of gully erosion. The vegetation coverage in the whole dry-hot valley area of Yuanmou is dominated by herbs with low vegetation coverage, although some places reached middle vegetation coverage [67]. Tutujiliangzi is in the vertisol area where the gully vegetation structure is single, the vegetation coverage level is low, and gully erosion activity is strong and mostly in the active stage. Shadi is in the dry red soil area where the vegetation coverage is relatively high, which is better than that in the vertisol area [67]. The development rate of the gullies is slower than that of Tutujiliangzi. In Jinlei, where the vegetation coverage is similar to Tutujiliangzi, the vegetation coverage in the gully system is extremely low and the surface cracking is serious, which accelerates gully development. In Maoyi, where the vegetation coverage is similar to Shadi, the vegetation coverage in the gully system is at a medium level, and the gully development is relatively slow.

## 5. Conclusions

Based on ArcGIS and UAV aerial survey data, this article extracted gullies and gully evolution indices of Tutujiliangzi and Shadi in Yuanmou County, by using the multi-criteria decision analysis method and the hypsometric integral method. The gully developmental stages were quantitatively analyzed from the aspects of the longitudinal profile, the cross profile and the knickpoint. Through the analysis of the longitudinal profile and the fitting function of 50 gullies in the study area, it can be concluded that: (1) Tutujiliangzi and Shadi are dominated by gullies in the deep incision period and the equilibrium adjustment period, which correspond to the gully head cutting stage and the equilibrium profile stage of gully development, respectively, and most gullies are in the active stage. This conclusion is verified by the shape fitting of the gully longitudinal profile and discrimination of the actual longitudinal profile shape, as well as the comparison with the previous research results. (2) The longitudinal profile morphological indices of 13 gullies are 0.636~0.933, showing an upward convex shape, and the cross profiles are mainly V-shaped and U-shaped. The longitudinal profile morphological indices of 32 gullies are 1.005~2.384, showing a slightly downward concave shape, and the cross profiles are mainly repeated U-shapes. The longitudinal profile morphological indices of 5 gullies are 0.592, 0462, 1.061, 1.344 and 0.888, respectively, and the morphological characteristics of upstream and downstream are different. (3) The gully longitudinal profile in Tutujiliangzi is generally close to a straight line and slightly concave. The gully longitudinal profile in Shadi is generally convex and close to a straight line, indicating that the gullies in the study area are in a period of intense erosion. (4) The knickpoints and step-pools are more developed, and the gullies of Tutujiliangzi develop more rapidly, which shows the influence of local topographic changes, soil properties and vegetation conditions on gully development.

A multi-criteria framework for the study of gully development stages has been established. This framework combined the research methods from different perspectives and the gully development stage was studied from three aspects: the longitudinal profile, the cross profile and the knickpoint. The results show that this framework can indeed be used in the study of gully development. Due to the continuous erosion and deposition during the development of gullies, which have a great influence on gully morphology, how gully erosion/deposition can be effectively applied to the investigation needs to be further explored. This study revealed that UAV photogrammetry used in the study of erosion gullies has the characteristics of high precision and timeliness. This experiment is helpful to improve the understanding of gully morphology and the development characteristics of gullies and provides a basis and reference for subsequent research on the erosion and deposition of gullies. In addition, the formation of gullies is a result of a combination of human activities and the natural environment and other factors, and the understanding of the influencing factors of gully formation, covered in the discussion section, needs to be improved. Therefore, it is an important direction for the future study of gully erosion to study the development stage of gully erosion combined with gully erosion/deposition and to study the influencing factors of gully morphology from the perspective of multifactor coupling.

## Figures and Tables

**Table 1:** Quantitative indices of the gully erosion development stage.

Evolution Period	Longitudinal Profile Morphological Index	Longitudinal Profile Information Entropy	Longitudinal Profile Shape	Developmental Stage	Terrain Features
Initial stage	N < 1	H(N) < 0.193	Convex	Deep incision erosion stage	Downward erosion is deepened, headward erosion is slowed down
Medium stage	N = 1	H(N) = 0.193	Close to a straight line	Transition stage	Gully bed expansion is slowed down
Late stage	N > 1	H(N) > 0.193	Concave	Equilibrium adjustment stage	Downward erosion is weak, wide gully develops
Terminal stage	N > 1	H(N) > 0.193	Concave	Equilibrium profile stage	Downward stopped and lateral erosion prevailed

**Table 2:** The gully index of Tutujiliangzi and the optimum-fit functions.

Gully Number	A (m^2^)	L (m)	N	H(N)	Morphology of Longitudinal Profile	Linear Function (R^2^)	Exponential Function (R^2^)	Logarithm Function (R^2^)	Power Function (R^2^)
T1	16.37	134.71	1.147	0.230	concave	0.983	0.962	0.944	0.995 *
T2	111.17	898.51	1.075	0.212	concave	0.732	0.803	0.477	0.916 *
T3	18.77	154.93	2.384	0.515	concave	0.815	0.636	0.881 *	0.740
T4	30.76	249.00	0.592	0.093	knickpoint	0.406 *	0.298 *	0.283	0.203
T5	18.74	145.47	0.462	0.064	knickpoint	0.989 *	0.996 *	0.977	0.987
T6	10.67	119.46	2.148	0.464	concave	0.964	0.954	0.854	0.999 *
T7	118.44	969.61	1.255	0.257	concave	0.884	0.868	0.733	0.972 *
T8	73.59	616.76	1.309	0.270	concave	0.823	0.933 *	0.671	0.944 *
T9	20.54	171.94	1.491	0.314	concave	0.942	0.992 *	0.846	0.978
T10	30.48	259.75	1.375	0.286	concave	0.999 *	0.989	0.985	0.999 *
T11	25.96	228.24	1.445	0.303	concave	0.970	0.831	0.996 *	0.967
T12	62.45	505.19	1.190	0.240	concave	0.790	0.934 *	0.624	0.856
T13	22.67	190.2	0.700	0.118	convex	0.739 *	0.634	0.529	0.433
T14	15.59	131.33	1.605	0.341	concave	0.998	0.975	0.969	0.999 *
T15	28.24	228.44	1.005	0.194	concave	0.880	0.967 *	0.682	0.905
T16	31.68	254.65	1.865	0.402	concave	0.962	0.971	0.835	0.996 *
T17	23.33	190.72	0.700	0.118	convex	0.423	0.441 *	0.246	0.271
T18	61.80	511.65	1.576	0.335	concave	0.711	0.924 *	0.508	0.920
T19	35.10	301.38	1.543	0.327	concave	0.761	0.898	0.602	0.910 *
T20	34.45	279.67	1.752	0.376	concave	0.925	0.996 *	0.786	0.976
T21	39.34	322.58	1.607	0.342	concave	0.880	0.984 *	0.801	0.945
T22	22.87	184.58	1.024	0.199	concave	0.959	0.981 *	0.853	0.921
T23	25.61	207.11	1.278	0.262	concave	0.954	0.998 *	0.879	0.971
T24	28.76	230.68	1.600	0.340	concave	0.881	0.951 *	0.824	0.911
T25	12.21	100.58	1.287	0.364	concave	0.999 *	0.928	0.970	0.993
Average	36.78	303.49	1.337	0.279					

“*” represents optimum-fit functions of longitudinal profile.

**Table 3:** The gully index of Shadi and the optimum-fit functions.

Gully Number	A (m^2^)	L (m)	N	H(N)	Morphology of Longitudinal Profile	Linear Function (R^2^)	Exponential Function (R^2^)	Logarithm Function (R^2^)	Power Function (R^2^)
S1	20.13	165.73	1.511	0.319	concave	0.991 *	0.647	0.869	0.942
S2	16.23	128.55	0.933	0.176	convex	0.982	0.935	0.907	0.994 *
S3	26.60	219.15	1.253	0.256	concave	0.965 *	0.831	0.961	0.905
S4	13.19	106.10	0.645	0.106	convex	0.944	0.846	0.972 *	0.959
S5	9.04	72.22	1.306	0.269	concave	0.926	0.828	0.981 *	0.918
S6	20.78	166.68	1.061	0.208	knickpoint	0.969 *	0.805	0.945	0.965
S7	10.90	88.72	0.927	0.175	convex	0.989 *	0.977	0.896	0.991 *
S8	16.53	134.53	0.636	0.104	convex	0.986 *	0.987 *	0.892	0.945
S9	16.68	136.85	1.027	0.200	concave	0.981	0.987 *	0.897	0.990 *
S10	23.43	195.99	1.331	0.275	concave	0.884 *	0.643	0.659	0.498
S11	24.99	206.22	1.344	0.279	knickpoint	0.972 *	0.870	0.893	0.974 *
S12	25.70	202.75	0.888	0.165	knickpoint	0.968 *	0.865	0.772	0.966
S13	23.58	192.10	0.839	0.153	convex	0.976 *	0.843	0.956	0.973
S14	17.45	143.07	0.748	0.131	convex	1.000 *	0.983	0.971	0.999
S15	12.95	105.58	0.912	0.171	convex	0.984	0.853	0.940	0.997 *
S16	24.09	194.02	0.839	0.153	convex	0.975 *	0.973	0.870	0.949
S17	24.62	197.31	2.120	0.458	concave	0.958	0.968	0.840	0.998 *
S18	14.45	116.95	1.498	0.316	concave	0.998 *	0.917	0.981	0.989
S19	12.29	99.36	0.922	0.175	convex	0.938	0.830	0.970 *	0.936
S20	11.36	91.71	1.519	0.321	concave	0.948	0.998 *	0.899	0.981
S21	6.04	50.53	0.771	0.136	convex	0.993	0.925	0.970	0.999 *
S22	24.75	198.16	1.209	0.245	concave	0.978	0.884	0.987 *	0.982
S23	22.57	181.15	1.527	0.323	concave	0.948 *	0.657	0.943	0.762
S24	17.57	147.90	0.760	0.134	convex	0.790	0.938 *	0.406	0.647
S25	5.03	40.82	1.524	0.322	concave	0.997 *	0.936	0.943	0.996
Average	17.64	143.29	1.122	0.223					

“*” represents the optimum-fit functions of longitudinal profile.

**Table 4:** Characteristics of gully development in different developmental stages.

Longitudinal Profile Morphological Index	Cross Profile	Longitudinal Profile	Gully Head	Knickpoint
0.636 < N < 0.933	V-shape or U-shape	Convex or straight	Begins to cut down, the top forms a drop or cliff	Knickpoints prone to occur in gully bed
1.005 < N < 2.384	Repeated U-shape	Concave	The top drop is not obvious, forming a smooth curve	Knickpoints close to gully head

## Data Availability

The data presented in this study are available on request from the corresponding author.
